# Case Report: Not Your Typical Kidney Stone

**DOI:** 10.21980/J8GD2T

**Published:** 2021-01-15

**Authors:** Laura Kolster, Danielle Biggs

**Affiliations:** * Morristown Medical Center, Department of Emergency Medicine, Morristown, NJ

## Abstract

The patient is a 66-year-old male, with a history of kidney stones, who presents to the emergency department complaining of dull, right-sided flank pain radiating to the right testicle that began one hour prior to arrival. The patient had a computed tomography (CT) of the abdomen and pelvis without contrast, which demonstrated a right renal calculus with forniceal rupture. This case represents a rare complication of a typical Emergency Department complaint; therefore, clinicians should be aware of its clinical relevance.

**Topics:**

Flank pain, forniceal rupture, renal colic, CT scan.

**Figure:**
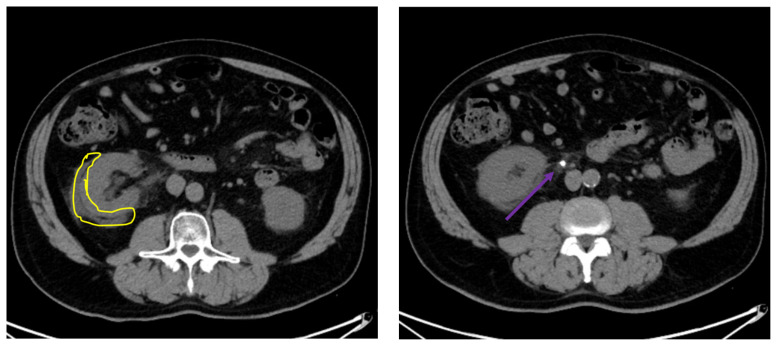

## Brief introduction

The diagnosis of forniceal rupture is a rare complication of nephrolithiasis. Urolithiasis is present in approximately 5–15% of the world population. The most likely risk factors for developing renal calculi are age, most commonly between 20–50 years old, male gender, and family history. In the United States, men experience renal calculi twice as commonly as women. 1 Forniceal rupture carries a high risk of abscess formation and/or urosepsis, both of which have a high mortality rate.

## Presenting concerns and clinical findings

The patient is a 66-year-old male who presents to the emergency department for dull, right-sided, flank pain radiating to the right testicle that began 1 hour prior to arrival. He states the pain woke him up from sleep, which prompted him to come to the ED. He reports a history of kidney stones and states that the pain is very similar to prior episodes. His last kidney stone was ten years ago on the left side. Patient did not take any medication for pain prior to arrival. The patient denies hematuria, dysuria, vomiting, or fever.

## Significant findings

The CT scan demonstrates nephrolithiasis with associated forniceal rupture. Encircled in the yellow outline is fluid, demonstrating a forniceal rupture. The stone is in the proximal aspect of the ureter, as highlighted by the purple arrow.

## Patient course

On physical exam, the patient appeared generally uncomfortable secondary to pain and had right-sided costovertebral angle tenderness to palpation. Otherwise, the remainder of his examination was unremarkable. His vitals were within normal limits. The patient had a creatinine of 1.2. He also had large blood in his urine without leukocyte esterase, nitrites, or any other signs of infection. Computed tomography was performed which revealed an obstructing 10mm renal calculus in the proximal aspect of the right ureter with forniceal rupture. The patient was treated with Ketorolac 15mg IV for pain and 1L normal saline bolus for hydration.

Urology was consulted in the Emergency Department. It was arranged that the patient could be discharged home, given that he was stable, and his pain was controlled. He would follow up with urology for stent placement urgently.

## Discussion

The most common cause of forniceal rupture is nephrolithiasis at approximately 73%, followed by cancer at 11%.^2^ Forniceal rupture most commonly occurs in males with an average age of 53 years.^3^ Stone size or location does not impact whether forniceal rupture will occur or not.^4^ A retrospective review found that stones causing forniceal rupture were in the proximal ureter in 24.3% of cases, distal ureter in 17.6% of cases and at the vesicoureteric junction in 58.1% of cases.^5^

In patients with forniceal rupture, there is a high risk of abscess formation and urosepsis, both of which carry a high mortality rate. However, conservative management is appropriate for those with uncomplicated cases. For those with complications such as persistent pain, fever, acute kidney injury, infection, sizable urinoma, and/or solitary kidney, intervention should be performed immediately. Intervention includes stent insertion, nephrostomy tube insertion, or urgent ureteroscopy with stone extraction. There is no clear indication to obtain advanced imaging for ureteral rupture such as a CT urogram because it would not change further management.

If a perinephric abscess was to occur, retrospective studies have shown a 12% mortality rate. Of the patients with abscess, 72% of those had a positive urine analysis and positive urine cultures.^7^ However, for those patients without signs of infection or acute kidney injury based on creatinine, discharge without antibiotics is appropriate management.^8^ Forniceal rupture is a rare complication of a common ED diagnosis.

## Supplementary Information










