# Supplementary figures and images for: Case Report: Not Your Typical Kidney Stone
Source: J Educ Teach Emerg Med. 2021 Jan 15;6(1):V12–4. doi: 10.21980/J8GD2T (PMC10332762; doi:10.21980/J8GD2T)

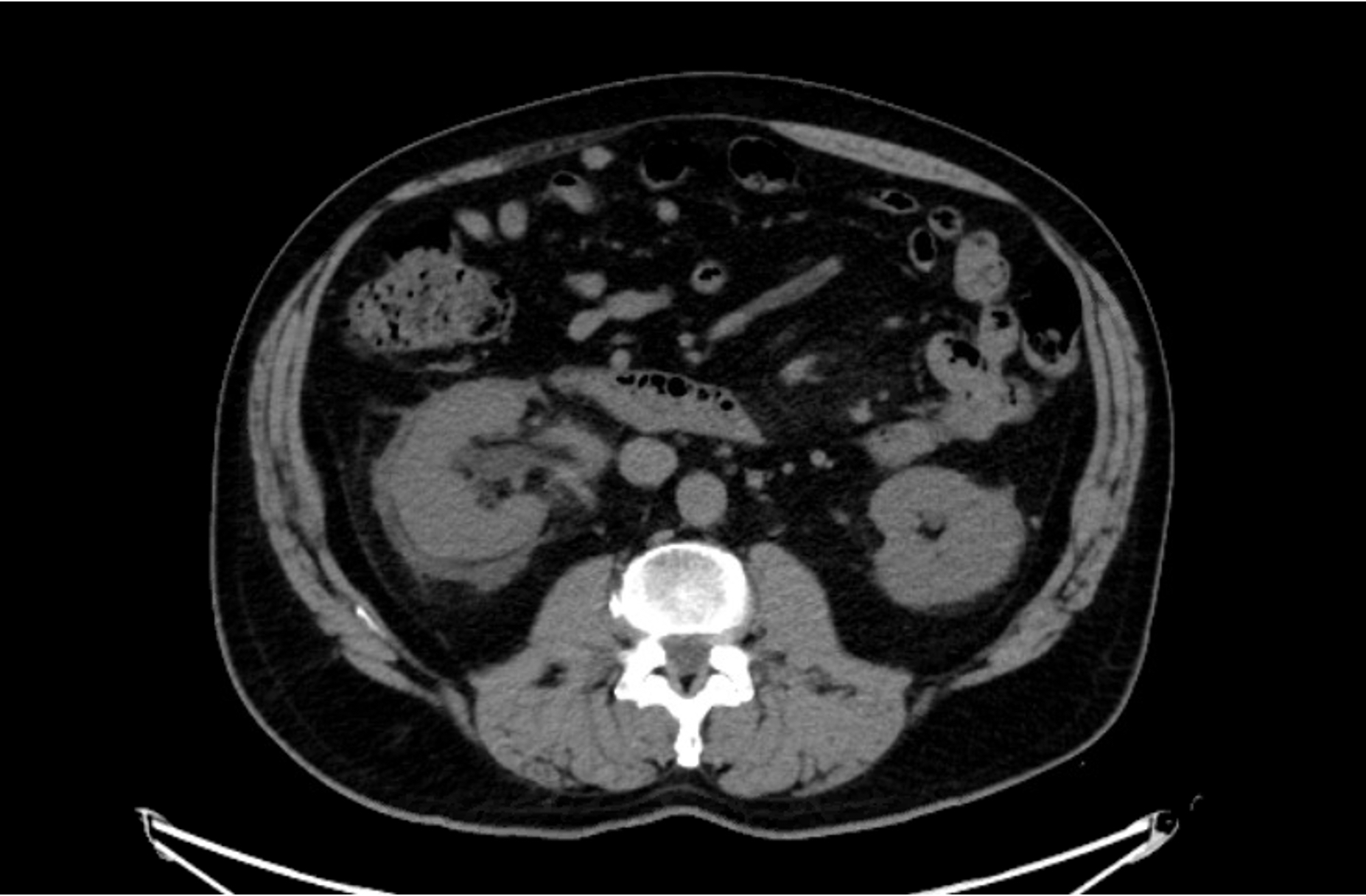

Supplement: Supplementary file 1 [file jetem-6-1-v12-supp1.jpeg]

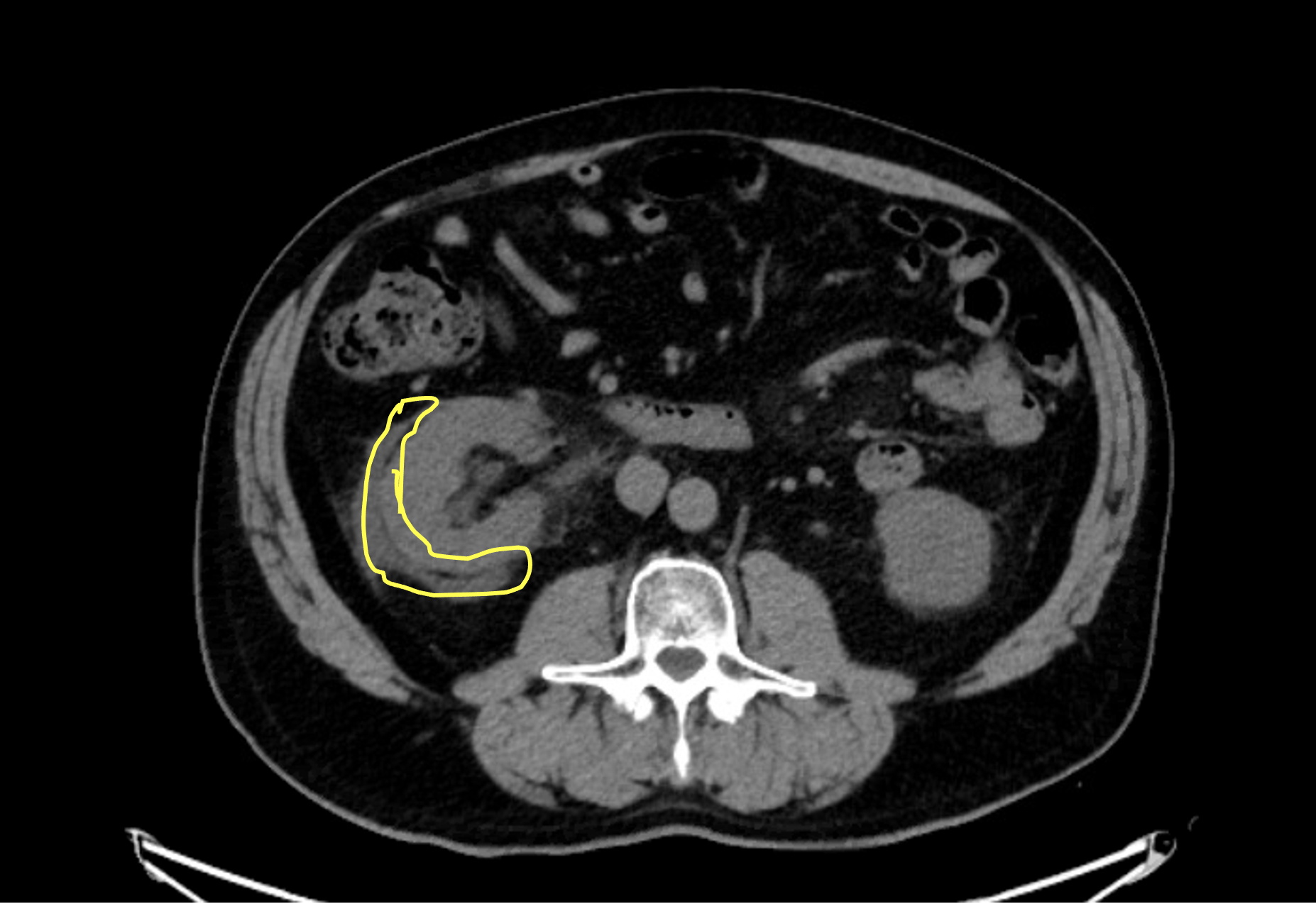

Supplement: Supplementary file 2 [file jetem-6-1-v12-supp2.jpeg]

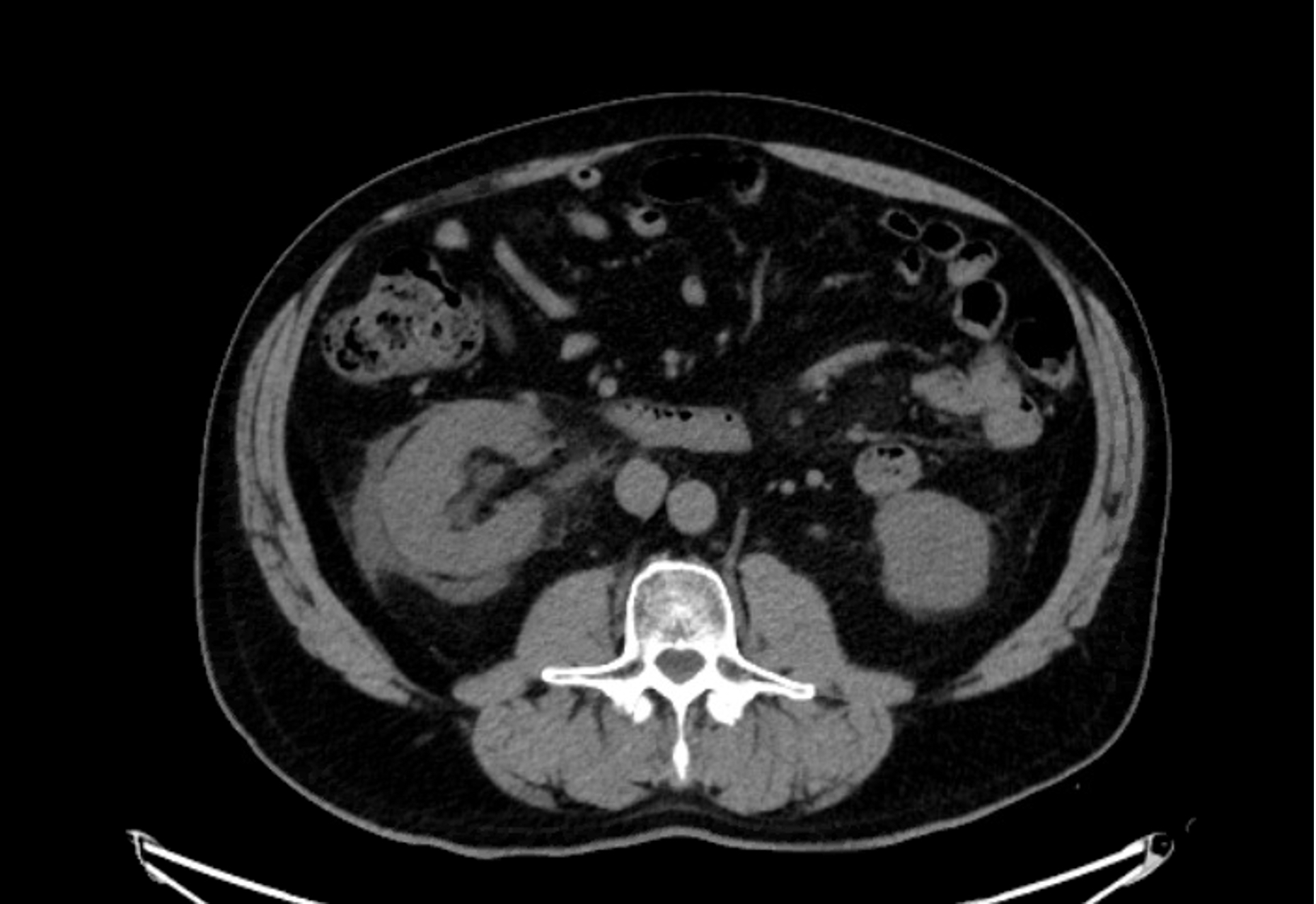

Supplement: Supplementary file 3 [file jetem-6-1-v12-supp3.jpeg]

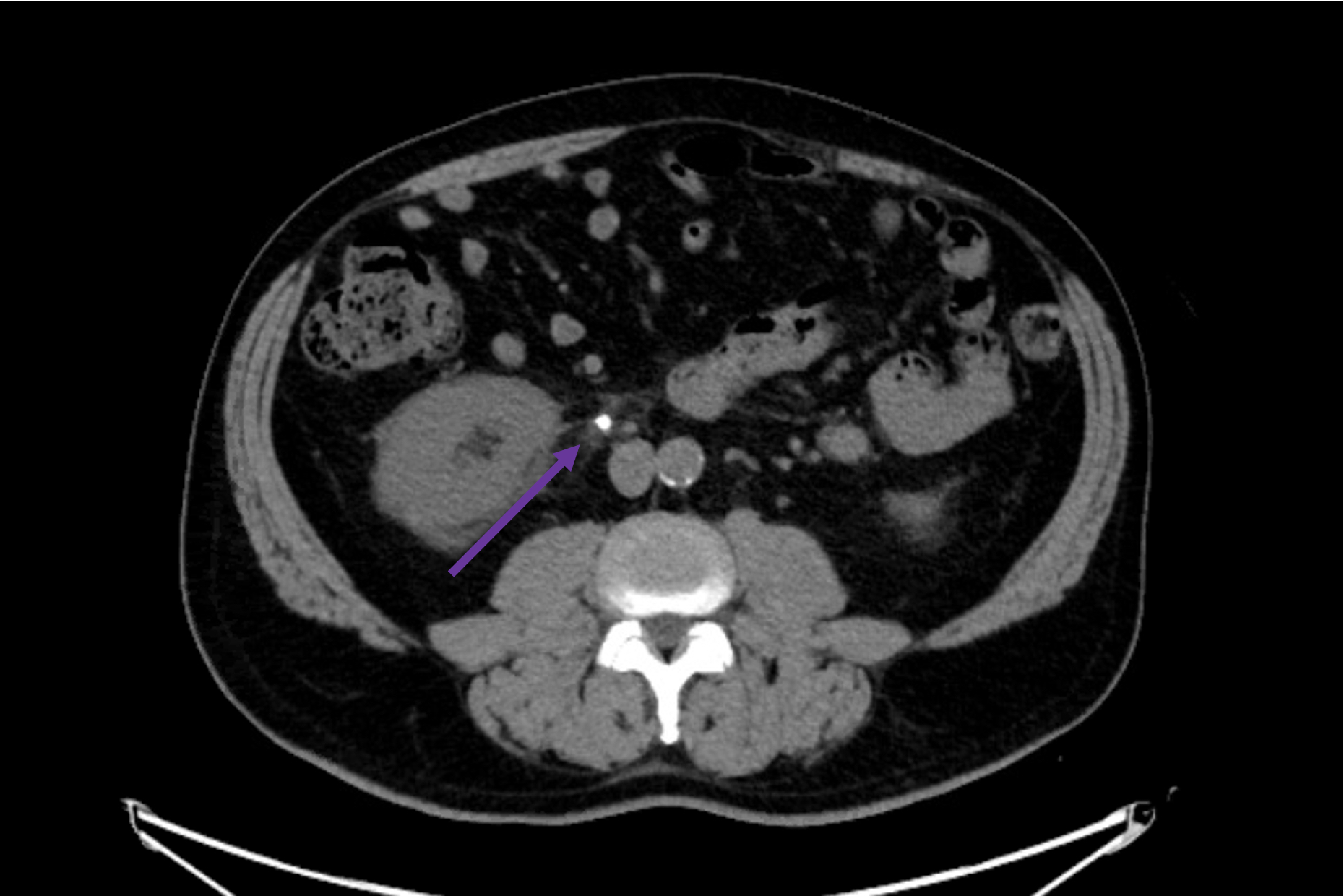

Supplement: Supplementary file 4 [file jetem-6-1-v12-supp4.jpeg]

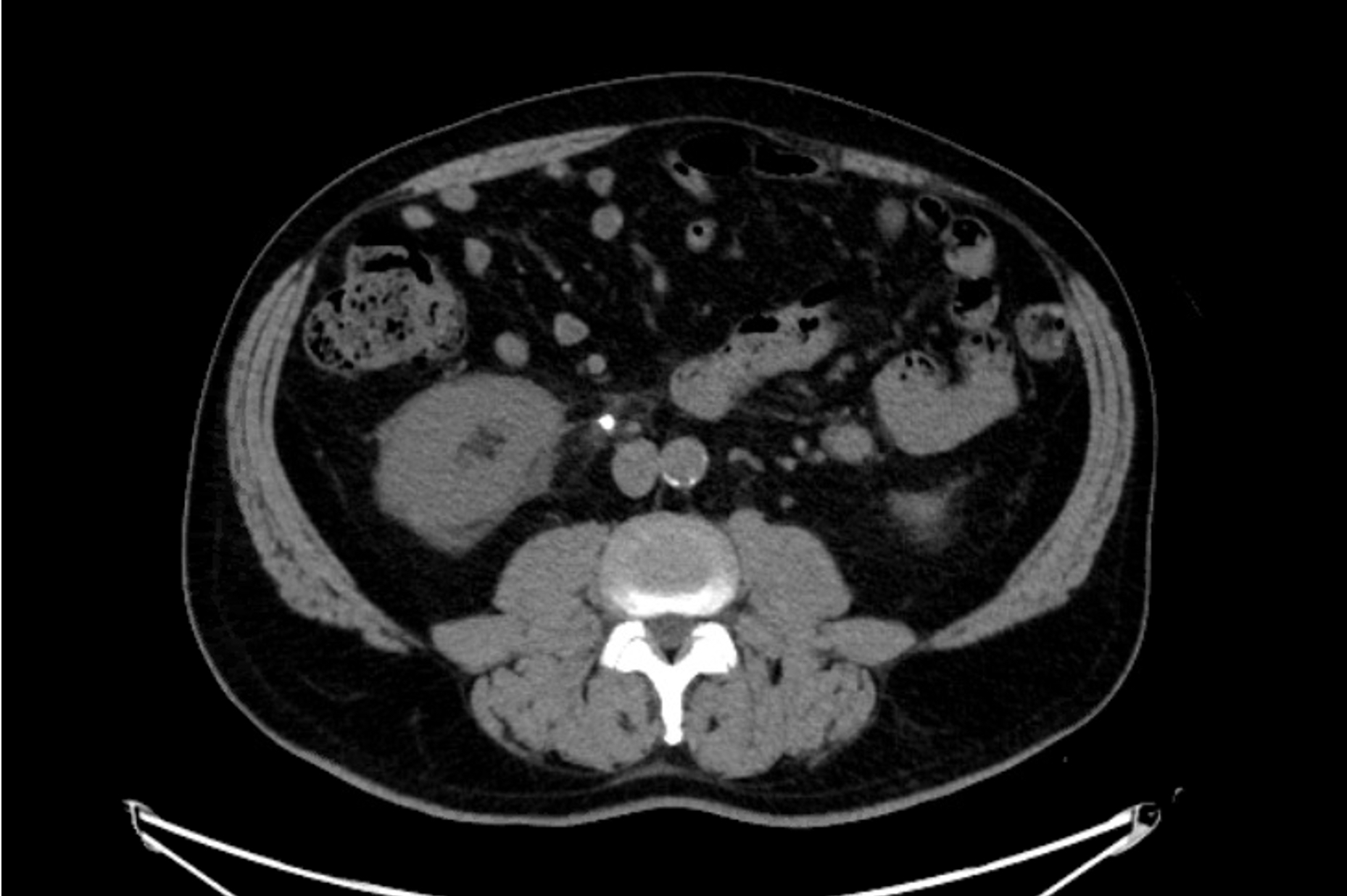

Supplement: Supplementary file 5 [file jetem-6-1-v12-supp5.jpeg]
